# Fungal community shifts in soils with varied cover crop treatments and edaphic properties

**DOI:** 10.1038/s41598-020-63173-7

**Published:** 2020-04-10

**Authors:** Mara L. Cloutier, Ebony Murrell, Mary Barbercheck, Jason Kaye, Denise Finney, Irene García-González, Mary Ann Bruns

**Affiliations:** 1 Department of Ecosystem Science and Management, Pennsylvania State University, University Park, 16801 PA USA; 2 Biogeochemistry Dual Title PhD Program–Pennsylvania State University, University Park, 16801 PA USA; 3 The Land Institute, Salina, 67401 KS USA; 4 Department of Entomology, Pennsylvania State University, University Park, 16801 PA USA; 5 Department of Biology, Ursinus College, Collegeville, 19426 PA USA; 6 Departamento de Producción Agraria, Universidad Politécnica de Madrid, Avda. Complutense s/n, 28040 Madrid, Spain

**Keywords:** Microbial ecology, Microbial communities, Environmental microbiology, Fungi

## Abstract

Cover cropping is proposed to enhance soil microbial diversity and activity, with cover crop type affecting microbial groups in different ways. We compared fungal community compositions of bulk soils differing by cover crop treatment, season, and edaphic properties in the third year of an organic, conventionally tilled rotation of corn-soybean-wheat planted with winter cover crops. We used Illumina amplicon sequencing fungal assemblages to evaluate effects of nine treatments, each replicated four times, consisting of six single winter cover crop species, a three-species mixture, a six-species mixture, and fallow. Alpha-diversity of fungal communities was not affected by cover crop species identity, function, or diversity. Sampling season influenced community composition as well as genus-level abundances of arbuscular mycorrhizal (AM) fungi. Cover crop mixtures, specifically the three-species mixture, had distinct AM fungal community compositions, while cereal rye and forage radish monocultures had unique Core OTU compositions. Soil texture, pH, permanganate oxidizable carbon, and chemical properties including Cu, and P were important variables in models of fungal OTU distributions across groupings. These results showed how fungal composition and potential functions were shaped by cover crop treatment as well as soil heterogeneity.

## Introduction

Microbial diversity is an important aspect of soil health, as soil microbial communities mediate many biogeochemical processes and are sensitive to disturbances that can lead to long-lasting ecosystem effects^1^. Greater richness and evenness in the representation of bacteria and fungi in soils can help mitigate plant responses to environmental stressors^2^. With the advent of high-throughput DNA sequencing technologies that allow for more detailed genetic information, we can determine if and how microbial compositions shift in response to disturbance and edaphic differences and, to some degree, what those changes may mean for ecosystem processes.

Cover cropping is the practice of growing ground-covering crops during the intervals between successive cash crops. Cover cropping imparts numerous benefits to soil, including the addition of organic carbon (C) from roots, root exudates, and aboveground residues and the improvement of soil structure and tilth^3–5^. Ecosystem services provided by cover crops include protection from soil erosion, enhanced soil water-holding capacity, reduced weed colonization and growth, plant resilience to pathogens and increased crop yield^5,6^. Legume cover crops provide biologically fixed nitrogen (N), while grasses take up excess soil inorganic N and improve N retention. A complete suite of ecosystem services is not deliverable by any one cover crop species. Thus, planting mixtures of cover crops has been proposed as a means to provide varied combinations of ecosystem services based on the functional traits of individual cover crop species^5,7–10^.

Cover crops may alter microbial community diversity and function by varying the types and composition of exuded C substrates from roots^5,11^. Fungi in agricultural soils possess an array of functional traits including degradative ability, plant infectivity, insect pathogenicity, and root symbiotic competence (e.g., arbuscular mycorrhizal, or AM fungi), in the subphylum Glomeromycotina^12^. Most studies of cover crop impacts on fungal communities have focused on AM fungi using either root colonization, spore extractions, or lipid biomarkers as assessment measures^13–20^. Increasingly, AM fungi are being studied using ribosomal RNA intergenic spacer (ITS) regions amplified from DNA extracts^21–25^. Overall, consistent patterns in cover crop species effects on AM fungal abundance and diversity have not been reported, with other factors such as soil type and season being more influential^26^.

As components of bulk soil microbial communities, mycorrhizal fungi make up a small, specialized fraction. To gain a broader understanding of cover crop effects on soil fungi overall, we used high-throughput DNA sequencing (Illumina MiSeq) of ITS regions to assess abundances of “core’ and ‘rare’ fungal taxa, in addition to mycorrhizal fungi and another specialized fungal group, the insect pathogens. Core microbiomes consist of taxa consistently found in communities from similar habitats^27^, and they are presumed to be representative and well-adapted to those habitat conditions. Core microbiomes in bulk soils of agroecosystems may be less sensitive to changes in management practices than non-core members of the microbiomes^28^. Identifying members of core assemblages for use as inoculants has been proposed to improve ecosystem functions of agricultural soils^29,30^. On the other hand, rare members of microbial communities (which we define as <0.1%) are not detected consistently across similar habitats, but they can represent sources of genetic diversity or generate pulses of activity in response to specific environmental cues^31,32^.

In this study, we investigated the impact of cover cropping with monocultures and polycultures on AM fungi, Insect Pathogens, Core, Rare, Abundant, and all fungal OTUs. To the best of our knowledge, different functional and abundance-based groups of fungal communities in bulk soils under various cover crop treatments have not been reported. We performed modeling to determine the most important soil physico-chemical variables in shaping composition within the fungal groups. Fungal communities were probed with an Illumina MiSeq using the fungal ITS regions from soil DNAs extracted from nine different cover crop treatments at a single field site in Central Pennsylvania^9^. At this field site, insect sentinel assays had revealed lower abundances of the entomopathogenic fungus, *Metarhizium robertsii*, under brassicas than under legume cover crops^33^. Therefore, we aimed to 1) assess the impact of cover crops on alpha-diversity, 2) determine if cover crops impact beta-diversity of the different fungal groups, 3) evaluate the impact that cover crops had on genera assigned to AM fungi and Insect Pathogen groups, and 4) identify soil physico-chemical variables that correlated with composition of the fungal groups. To facilitate these analyses, we grouped cover crops by species (CC; Canola, Clover, Oat, Pea, Radish, Rye, 3Spp, 6Spp, and Fallow), by cover crop functional type (CC Function; Legume, Grass, Brassica, Mixture, and Fallow) or cover crop diversity (CCD; 0Spp, 1Spp, 3Spp, 6Spp).

## Results

### Fungal community characteristics

Fungal communities in bulk soil samples from nine cover crop treatments were characterized using ITS-based OTU groupings (four replicates at both spring and summer sampling times). Rarefaction curves of the fungal OTUs across the 72 samples are shown in Supplementary Fig. 1, and Supplementary Table 1 shows general statistics regarding each sample. After removing singletons, 16 682 OTUs were observed from all 72 samples, with 12 275 OTUs taxonomically classified as fungal sequences. Of the abundance-based groupings, most OTUs were rare (11 358 out of 12 275, or 92.5%), but these accounted for only a small fraction (6.1%) of total fungal sequences (Table 1). In contrast, abundant OTUs made up 7.5% of total fungal OTUs but accounted for 86–99% of all fungal sequences. Similarly, the 276 core OTUs (found in all samples) made up only 2.2% of total fungal OTUs but represented 45.8–95.7% (average of 79%) of fungal sequences in the samples. Absolute abundances and relative abundances for each OTU grouping based on non-rarefied samples can be found in Supplementary Table 2.

**Table 1:** Total OTU counts in functional and abundance-based groupings and % of all reads that each grouping accounts for.

Grouping Type	OTU Grouping	Abundance	Number of OTUs	% of Total OTUs (12275)	Average % of Total Reads
Functional Grouping	Insect Pathogens	All	114	0.9	0.168
		Rare only	114	0.9	
		Abundant only	0	0	
	AM fungi	All	646	5.3	3.332
		Rare only	639	5.2	
		Abundant only	7	0.01	
Abundance Grouping	Rare		11358	92.5	6.064
	Abundant		917	7.5	93.832
	Core		276	2.2	78.951
	All		12275	100	100

Functional groupings are also divided into “rare” and “abundant” based on whether the OTU was <0.1% abundant across all samples.

At the phylum level, OTUs were classified predominantly as Ascomycota or Basidiomycota, accounting for 34–57% and 18–55%, respectively, of fungal sequences across samples (Supplementary Fig. 2). At the class level, Agaricomycetes had the highest overall abundance followed by Sordariomycetes and Mortierellomycotina (Supplementary Fig. 2). At the OTU-level, OTU 1 was assigned to *Coprinus* spp. and had the highest total abundance, accounting for 0.4–27% of fungal sequences per sample. The next two most abundant OTUs were assigned to *Mortierella* (OTU 13 032 and 7 311; Supplementary Fig. 3). *Mortieriella* was the most abundant genus followed by *Coprinus*, accounting for 10.9–23.4 and 0.4–33.3% of the fungal sequences.

Most OTUs in the two functional groupings, AM fungi and Insect Pathogens, were classified as rare, being found at levels of <0.1% across all samples (Table 1). Out of 12 275 total OTUs, only 114 (0.9%) were assigned to the potential Insect Pathogen group, while 646 OTUs (5.3% of total OTUs) were classified as Glomeromycotina and grouped as AM fungi (Table 1). When tallied as percentages of total sequences, Insect Pathogens and AM fungi comprised approximately 0.2% and 3.3% of all sequences, respectively (Table 1).

### Genera-level changes by CC, function, or season

#### AM fungi

**Figure 1:**
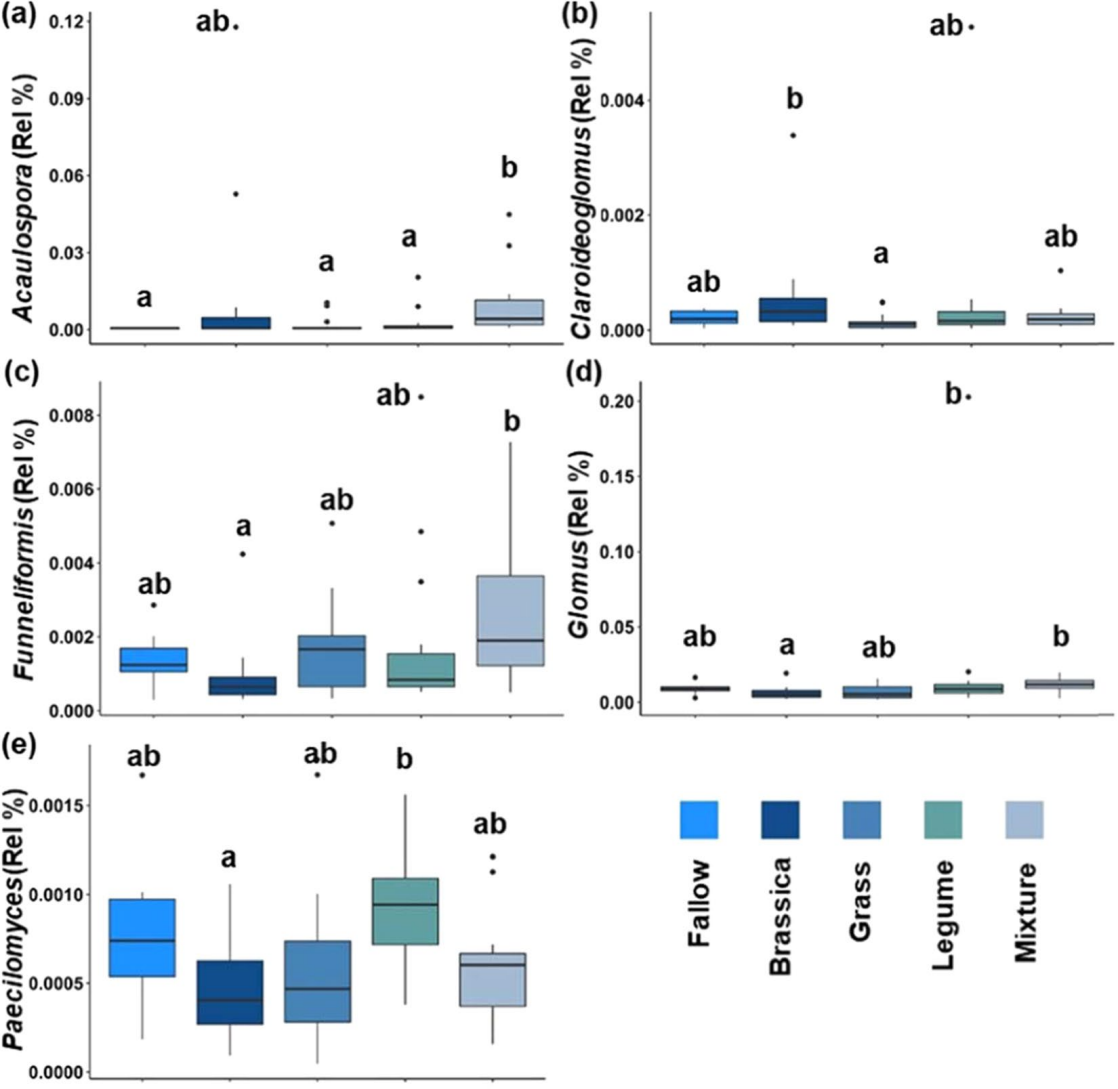
Relative abundances of genera across CC Function. Genera classified as AM fungi (**a**–**d**) or as an Insect Pathogen (**e**). Lower case letters denote significant differences among Functions (p < 0.05; n = 8 for Fallow, n = 16 for Brassica, Grass, Legume, and Mixture). Circles represent outliers, the horizontal lines represent the median values, and the vertical lines represent the minimum and maximum values excluding outliers.

**Figure 2:**
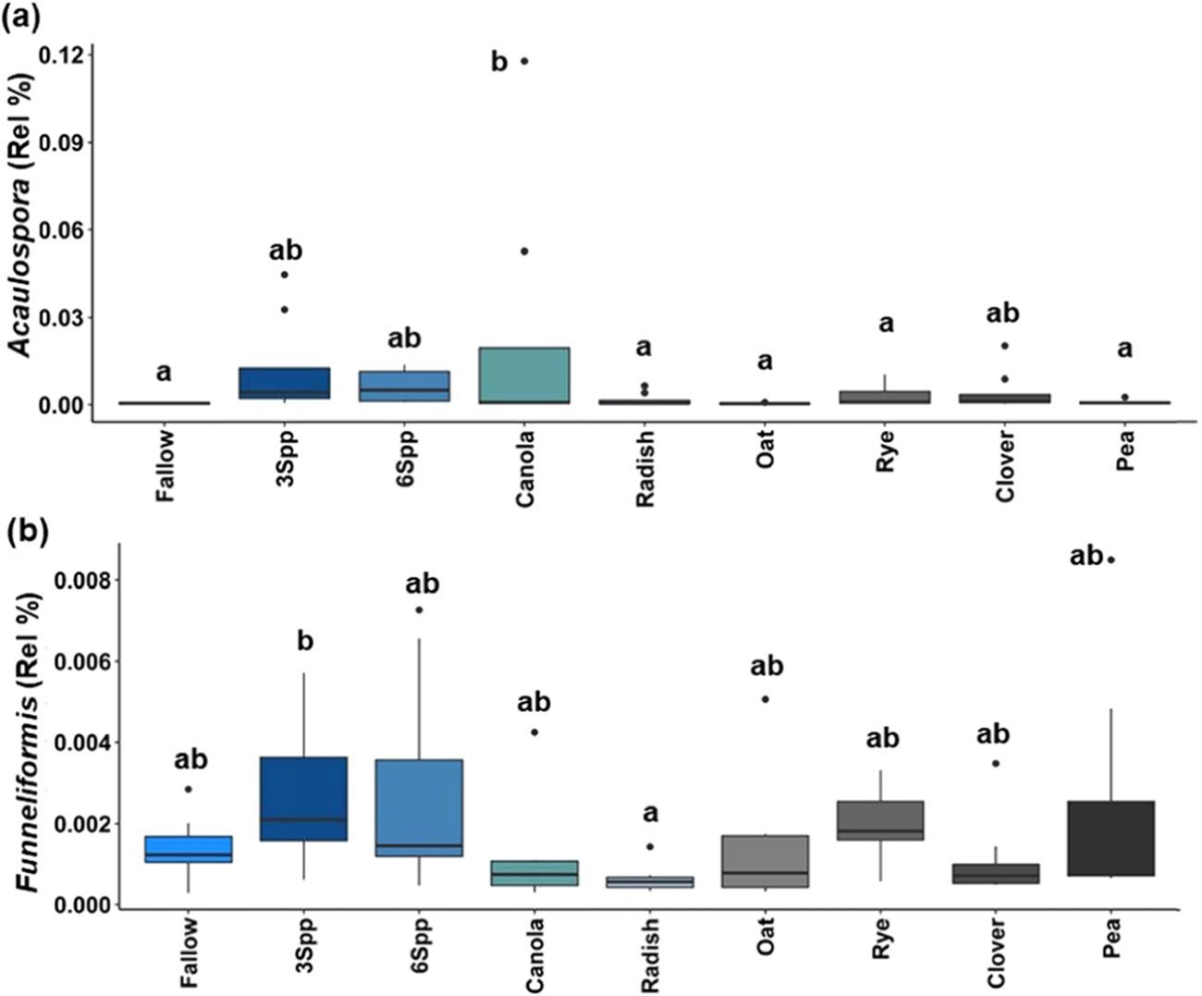
Relative abundances of genera across CC. Both genera, *Acaulospora* (**a**) and *Funneliformis* (**b**) were assigned to the AM fungal group. Lower case letters denote significant differences among CC (p < 0.05; n = 8). Circles represent outliers, the horizontal lines represent the median values, and the vertical lines represent the minimum and maximum values excluding outliers.

**Figure 3:**
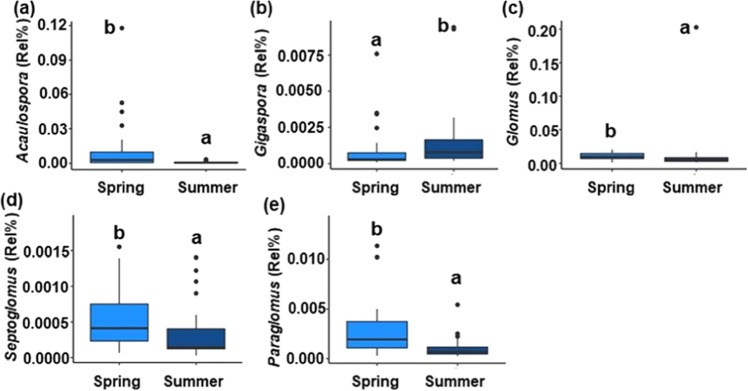
Relative abundances of genera in the AM fungal group by Season. Lower case letters denote significant differences between Time (p < 0.05; n = 36). Circles represent outliers, the horizontal lines represent the median values, and the vertical lines represent the minimum and maximum values excluding outliers.

At the genus-level, CC Function, CC, and Season, were significant factors in explaining the abundances of several genera of AM fungi (Table 2). Abundances of four AM fungal genera were related to CC Function (Table 2). Mixtures supported higher abundances of *Acaulospora* compared to other CC Functions except for Brassicas, while Brassicas had significantly lower abundances of *Glomus* and *Funneliformis* compared to Legumes and/or Mixtures but higher abundance of *Claroideoglomus* compared to Grasses (Fig. 1). *Acaulospora* and *Funneliformis* were the only genera affected by CC (Table 2). *Acaulospora* was highest in Canola compared to Fallow, Oat, Pea, Radish, and Rye treatments and *Funneliformis* was higher in the 3Spp mixture compared to the Radish treatment (Fig. 2). Season was also a significant factor when assessing abundances of AM fungal genera (Table 2). Of the 10 genera analyzed from the AM fungal grouping, five had different abundances across the spring and summer samples. *Gigaspora* was the only genus that was higher in the summer compared to spring, while all others were higher in the spring (Fig. 3).

**Table 2:** Genus-level linear mixed models of genera assigned to the AM fungi and Insect Pathogens group across cover crop (CC), cover crop and season (CC & Season), steason, plant functions (Function), and plant functions and time (Function & Season).

	Genera	CC	CC & Season	Season	Function	Function & Season
AM fungi	*Acaulospora*	0.012*	0.084	0.006*	0.012*	0.434
	*Claroideoglomus*	0.134	0.784	0.706	0.016*	0.449
	*Entrophospora*	0.378	0.784	0.434	0.980	0.545
	*Funneliformis*	0.049*	0.784	0.418	0.016*	0.495
	*Gigaspora*	0.793	0.784	0.014*	0.584	0.454
	*Glomus*	0.134	0.784	0.014*	0.016*	0.694
	*Paraglomus*	0.587	0.784	0.006*	0.394	0.454
	*Rhizophagus*	0.378	0.784	0.370	0.125	0.965
	*Scutellospora*	0.501	0.784	0.614	0.364	0.449
	*Septoglomus*	0.517	0.812	0.010*	0.305	0.495
Insect Pathogens	*Metarhizium*	0.134	0.459	0.370	0.067	0.434
	*Paecilomyces*	0.131	0.784	0.370	0.016*	0.454

Significant p-values are denoted with an *(n = 8 for CC, n = 4 for CC* Season, n = 36 for Season, n = 8/16 for Function; n = 4/8 for Function* Season).

#### Insect Pathogens

Of the four genera represented in the Insect Pathogen grouping, *Metarhizium* and *Paecilomyces* were sequenced from all of the samples. *Beauveria* spp. and *Ophiocordyceps sinensis* were detected in less than 50% of samples and, thus, were not included in the linear mixed model analyses. *Metarhizium* abundance was not different between the CC’s or Function, while *Paecilomyces* was affected by Function (Table 2; p < 0.05). Further inspection indicated that *Paecilomyces* was higher in abundance in the Legumes compared to the Brassicas (Fig. 1; p < 0.05).

### Fungal assemblage ecological metrics

Across cover crop treatments, fungal alpha-diversity based on the Shannon diversity index ranged between 3.20 and 5.25. Alpha-diversity based on richness values ranged between 1 881 and 2 703 (Supplementary Table 3). No differences were observed in richness based on cover crop diversity (CCD; 0, 1, 3, 6 Spp.), CC, Function, Season, or any interactions (p > 0.05; Supplementary Table 4). Shannon diversity index did not differ by CCD, CC, Function or any interactions, but they did differ by Season (p < 0.05). In the spring, the Shannon index was higher (4.75) compared to the summer (4.23) (p < 0.05).

**Figure 4:**
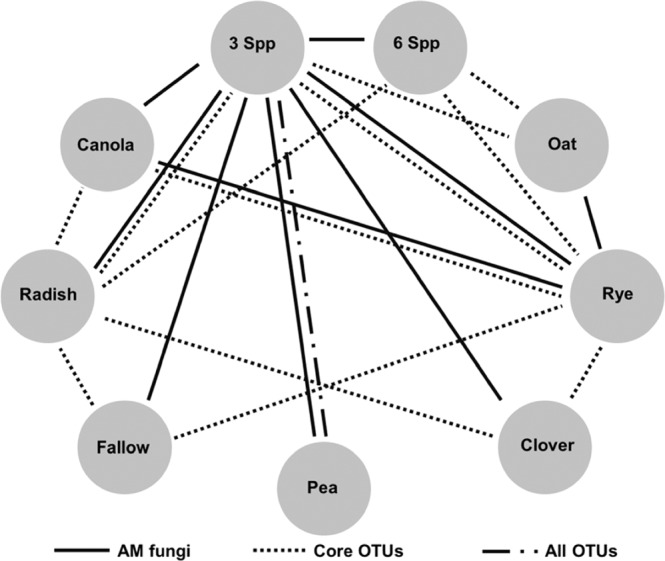
Relational diagram showing differences in fungal community compositions across CC. Lines connecting CC’s indicate differences (p-value <0.05) calculated by pairwise PERMANOVA comparisons using the Bray-Curtis dissimilarity matrix (n = 4) and the RVAideMemoire package in R.

Fungal community composition differed between the spring and summer sampling events for every OTU grouping (Table 3). Compositions also differed significantly by CC for AM fungi, Rare, Core and All OTU groupings, but no interactions were observed between CC*Season for any grouping (Table 3). Similarly, AM fungi, Rare, Core, All, and Insect Pathogens had different fungal compositions when assessing the importance of Function but not when assessed by Function*Season (Supplementary Table 4). Some patterns emerged and are shown in relational diagrams (Fig. 4 and Supplementary Fig. 4). Overall, the most notable differences across CC Functional groups were observed for the Core OTUs. Mixtures had significantly distinct Core OTU compositions compared to Brassicas, Legumes, and Grasses, while Grasses were distinct from Mixtures, Legumes, and Fallow (Supplementary Fig. 4). Composition of AM fungi in the Mixtures differed from those in Grasses, Legumes, and Fallow treatments. Insect pathogen composition also differed between Grasses and Legumes (Supplementary Fig. 4). It should be noted that the differences observed between the Mixtures and other Functions seem to be mainly driven by the 3Spp mixture (Fig. 4). Two other groupings, Rare and All, also showed significant differences in the initial PERMANOVA (Supplementary Table 5; p < 0.05) but no pairwise comparisons were significant following p-value adjustments (p > 0.05).

**Table 3:** Results from PERMANOVA using Bray-Curtis dissimilarity for each OTU grouping by CC and Season (n = 8 for CC; n = 36 for Season, n = 4 for CC*Season).

OTU group	Factors	DF	SumSqs	MeanSqs	F.model	R^2^	Pr(>F)
AM fungi	CC	8	2.426	0.303	1.414	0.146	0.003*
	Season	1	0.975	0.975	4.546	0.059	0.001*
	CC* Season	8	1.696	0.212	0.989	0.102	0.514
	Residuals	54	11.579	0.214			
Insect Pathogens	CC	8	1.167	0.146	1.121	0.123	0.278
	Season	1	0.303	0.303	2.328	0.032	0.043*
	CC* Season	8	1.025	0.128	0.985	0.108	0.519
	Residuals	54	7.023				
Abundant	CC	8	1.494	0.187	0.914	0.103	0.792
	Season	1	0.511	0.511	2.499	0.035	0.006*
	CC* Season	8	1.480	0.185	0.905	0.102	0.816
	Residuals	54	11.039				
Rare	CC	8	2.603	0.325	1.169	0.127	0.001*
	Season	1	0.769	0.769	2.764	0.038	0.001*
	CC* Season	8	2.070	0.259	0.929	0.101	0.969
	Residuals	54	15.032				
Core	CC	8	1.9346	0.2418	1.4925	0.150	0.005*
	Season	1	0.908	0.9080	5.6039	0.071	0.001*
	CC* Season	8	1.2755	0.1594	0.9841	0.099	0.485
	Residuals	54	8.7494				
All	CC	8	2.1371	0.2671	0.3954	0.144	0.004*
	Season	1	0.9049	0.9049	4.7268	0.061	0.001*
	CC* Season	8	1.4891	0.1861	0.9723	0.100	0.576
	Residuals	54	10.3378				

Significant p-values are denoted with an*.

Across all CC, 3Spp and Rye supported more distinctive fungal compositions, exhibiting 11 and 8 significant pairwise differences, respectively, compared with other CC species (Fig. 4). Core OTU compositions were distinct in the Rye and Radish, each showing differences with Canola, Clover, Fallow, 3Spp, and 6Spp cover crop treatments. These five latter treatments, as well as Pea, all had similar core microbiome compositions (Fig. 4). Results based on simper analysis indicated that OTU 1 was driving the dissimilarities observed with Radish accounting for 12.6–18.4% of the total dissimilarity between Radish and 3Spp, 6Spp, Canola, Clover and Fallow (Supplementary Table 6). For Rye, OTU 1 and OTU 3 were among the top five most influential OTUs for driving the dissimilarities observed in Fig. 4. and when combined accounted for ~ 30% of the dissimilarities (Supplementary Table 6). Both OTUs (1 and 3) were in the top 20 most abundant OTUs across the samples (Supplementary Fig. 3) and identified as *Coprinus sp*. and *Rhizoctonia solani*.

Composition of the AM fungal group also showed patterns in that 3Spp differed from every other CC except for Oat. Composition of AM fungi in Rye differed from AM fungi in Canola, 3Spp, and Oat (Fig. 4). Further analysis revealed that two OTUs in the AM fungal group, OTU 154 and 91, assigned to *Acaulospora morrowiae* were ranked in the top five most influential OTUs driving dissimilarities between the 3Spp mixture and other CCs (Supplementary Table 7). In all comparisons OTU 154 contributed most to the dissimilarities and when combined with OTU 91 approximately 18.7–24.5% of the dissimilarity between the 3Spp mixture and the other CC’s was explained.

### Edaphic factors associated with fungal community structure

**Figure 5:**
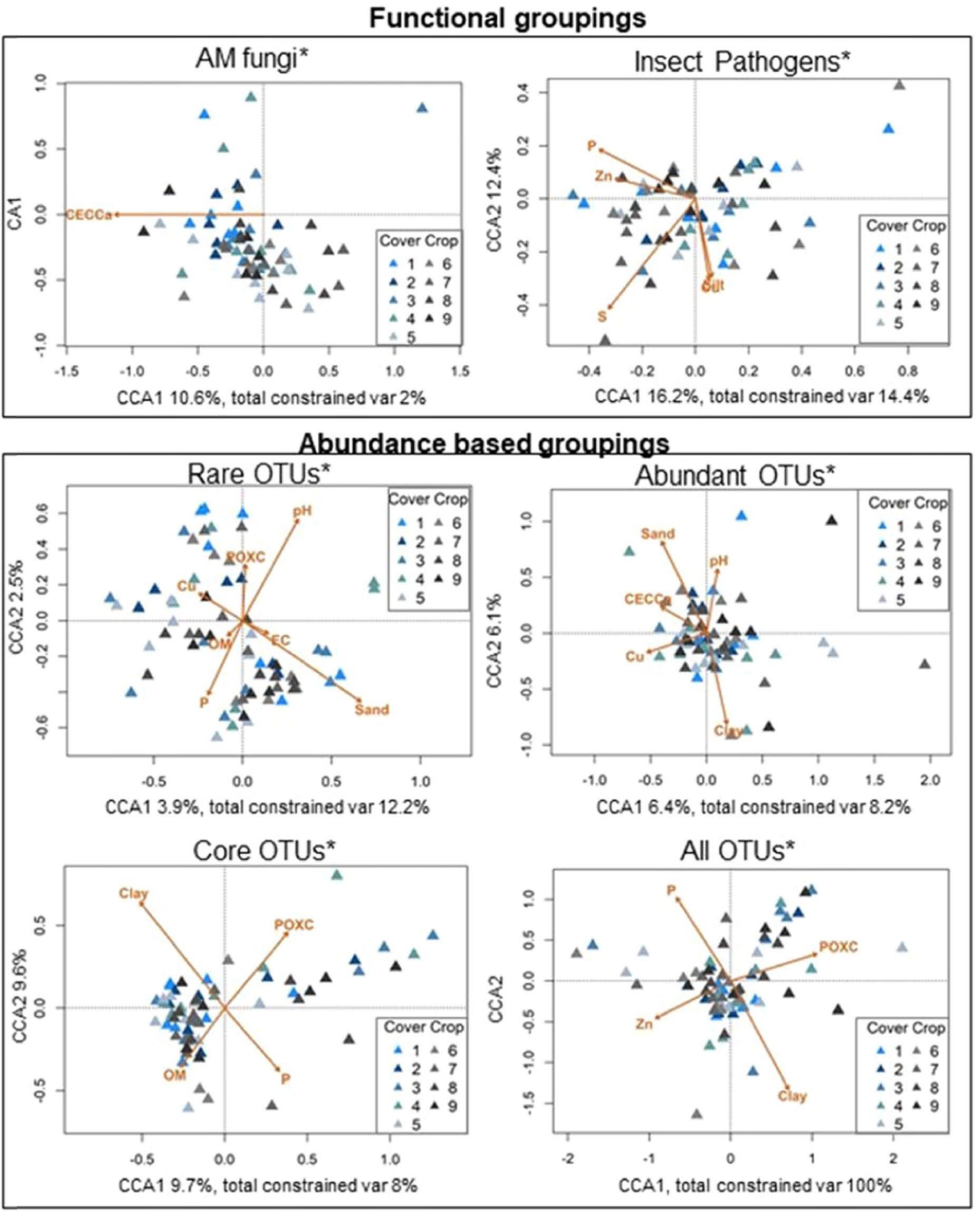
Constrained ordination plots using CCA models of OTU groupings. Important environmental variables selected using forward and backwards selection. (Supplementary Table 6). Each model that significantly explained the species dispersion across the OTU groupings are denoted with an *. Lengths of each vector indicate the correlation between the variable and the ordination. Percentages next to CCA1/CCA2 represent the amount of inertia explained by the axes, while total constrained var represents the amount of inertia accounted for by the vectors (environmental variables).

Over half of the soil physico-chemical variables were identified as significant contributors to modeling fungal compositions across samples (Table 4). Composition of AM fungi was best explained with only CEC-Ca while seven variables were needed to explain distributions observed for the Rare OTUs (sand, Cu, EC, P, pH, and permanganate oxidizable carbon (POXC)). Fungal compositions modeled with the environmental variables are plotted in Fig. 5. All six models explained significant portions of the compositions of fungal assemblages across the samples (ANOVA; p < 0.05). Of all six models, five of them included soil textural components, sand, silt, or clay. The model that explained the greatest amount of variation in fungal communities was observed for All OTUs, in which 100% of the variation in All OTUs was explained by Clay, P, POXC, and Zn, followed by Rare OTUs in which 16.1% of the variation was explained by the 7 environmental variables included in the model.

**Table 4:** Variables identified as contributing to the variation in species dispersions across the OTU groupings (+) using CCA and forward/backward selection to identify the best fitting models.

Environmental variable	AM fungi	Insect Pathogens	Rare	Abundant	Core	All	Total Significant OTU Groupings
CEC-Ca%	+			+			2
%Clay				+	+	+	3
%Sand			+	+			2
%Silt		+					1
Cu		+	+	+			3
EC			+				1
OM			+		+		2
P		+	+		+	+	4
pH			+	+			2
POX-C			+		+	+	3
S		+					1
Zn		+				+	2
Total Significant Contributors	1	5	7	5	4	4	

EC = electrical conductivity. OM = organic matter.

CEC, CEC-Mg, CEC-K, Ca, K, Mg, NH_4_^+^, NO_3_^−^, gravimetric water content, and matric potential were removed from the table because there were no groups that were significantly impacted by these variables.

## Discussion

Community composition in every OTU grouping shifted between spring and summer sampling times, including groupings not affected by cover crop species identity (i.e., Insect Pathogens and Abundant OTUs). Strong effects of season and/or time on fungal communities, especially AM fungi, from cover crop treatments have been observed elsewhere^34–37^. The two main disturbances that occurred between the spring and summer sampling events were the addition of bedded dairy manure and tillage. Studies of tillage practices comparing conventional moldboard plowing, conservation tillage such as chisel/disking, and no-till indicate that fungal communities are sensitive to the type of tillage practiced^38–40^.

*Mortierella* and *Coprinus*, the two most dominant fungal genera identified herein, are common inhabitants of manure and compost^41,42^ and have been found to dominate manured soils^43^. Since both plowing and manuring had been performed between the spring and summer sampling events, it is not possible to determine whether the effects on fungal communities observed were due to seasonal changes, tillage, or manure addition. Considering the potential importance of *Mortierella* in breaking down complex carbon sources, promoting plant growth, and improving soil health^44–46^, further exploration of factors influencing *Mortierella* growth in agricultural soils should be performed.

Compositions of all OTU groupings were significantly impacted by soil physico-chemical properties, including Cu, P, and Zn, pH, POXC, and soil texture. Copper-based fungicides had been used on a previous tomato experiment that overlapped with the area occupied by several plots in this study, thereby affecting their Cu concentrations. Fungal compositions in the Insect Pathogen, Abundant and Rare OTU groupings were all significantly related to Cu concentrations across the experimental plots. Copper can impact bacterial alpha- and beta- diversity^47^, constrain fungal community responses to carbon additions^48^, and alter microbial enzyme activities^49^. Results from these studies, combined with our present findings, suggest that Cu-based fungicides may have the potential for long-lasting (at least three years) impacts on fungal functions in soil.

Soil texture has been implicated as an important factor in shaping microbial communities in vegetable-cover crop rotations in Oregon^50^, in arable soils in Australia^51^, and in fungal phylogenetic relationships in a tillage experiment conducted in northern China^40^. Soil textural components, sand, stilt or clay, were identified as important variables in all of the CCA models with the exception of AM fungi. Given the importance of soil texture in shaping microbial communities within this study, it is not surprising that soil texture has been hypothesized to be a primary driver of microbial community structure^51^. Still, potential limitations of soil texture in supporting plant-microbe interactions remain to be explored. Interestingly, the individual field site in this study, which appeared to have homogenous tilth, had enough textural variation to affect fungal communities. Because soil texture is an inherent soil property, the shaping of fungal community composition by soil texture could affect fungal responsiveness to management practices, such as those intended to increase proliferation and root colonization by AM fungi or support fungal insect pathogens.

### Insect pathogenic genera

Of the four genera identified as potential insect pathogens, only two were found in at least 50% of all plots sampled. Greater detection of *Metarhizium* across the plots sampled compared to *Beauveria* and *Ophiocordyceps* across plots may be due to the ability of *Metarhizium* to survive outside of insect hosts^52^. *Paecilomyces* abundance was dependent on CC Function, with higher abundances observed in the Legumes compared to the Brassicas. In a previous study at the same site, recovery of *Metarhizium* spp. using insect sentinel bait traps was greater in Legumes than in Brassica treatments^33^. Two possible explanations for differing abundances of the entomopathogens in Brassicas and Legumes exist, the first is that brassicaceous plants exude S-containing compounds that negatively affect entomopathogenic fungi^53,54^. The second explanation is that because *Paecilomyces* is a pathogen of pests that infect legumes^55,56^ greater abundance of *Paecilomyces* may result from higher densities of pests in the legume treatments. Although further testing is needed to assess more fully the impact of Brassicas and Legumes on Insect Pathogens, our results support the idea that cover crop species selection could be a management approach to manipulate insect-pathogenic fungi and manage belowground insect herbivory.

### AM fungal genera

Genus-level abundances of OTUs assigned to the AM fungal group were dependent on cover crop identity, as well as CC Function. Previous studies have reported that cover crop effects on AM fungi, including biomass and community composition, seem to be dependent on cover crop species identity^19,21,23,25,36–38,57–59^. Our study indicates that not only are the effects dependent on CC identity but they vary among genera of AM fungi. Abundances of *Funneliformis*, and *Glomus* were lower in Brassicas compared to either the Mixtures or the Mixtures and the Legumes, while the relative abundance of *Claroideoglomus* was higher in Brassicas compared to Grasses. Lower abundances of AM fungi were expected in Brassica treatments, as the glucosinolates exuded by Brassicas have been demonstrated to negatively affect AM fungal colonization^60^. Certain Brassicas including Canola have been found to have AM fungal partners, but even these fungal genera are constrained to *Gigaspora*, *Funneliformis*, and *Rhizophagus* (see review by^61^). Since Brassicas had higher AM fungal abundances than grasses but lower abundances compared to Mixtures and Legumes, it appears that the relationships between Brassicas and AM fungi are complex. Further exploration is required to fully understand the ability of cover crops to recruit and establish relationships with AM fungi.

### Composition of OTU groupings

Effects of cover crops on fungal community composition varied with the fungal grouping analyzed. Cover crop identity was most influential on Core OTUs and AM fungi, compared to Insect Pathogens, Rare, Abundant, and All OTUs. Core OTUs accounted for nearly 80% of all sequence reads but only a small portion of the actual OTUs identified, which is similar to findings from sugarcane^62,63^, rice and maize^64^. Given the consistent presence of Core OTUs across samples, their relative abundances were largely dependent on cover crop identity. Defining the function of the core microbiome and how that function changes in relation to compositional changes will be an important next step to address in agroecosystems^27^.

In contrast to the Core OTUs, in which two monocultures had the most unique community compositions (Radish and Rye), the most distinctive AM fungal communities were observed in the 3Spp mixtures comprising pea, clover, and rye. The AM fungal composition in 3Spp differed from all other cover crop treatments except for Oat, which was a species not included in the 3Spp treatment. Cover crop monocultures^24,59,60,65^ and polycultures^16,19^, can increase the AM fungal inoculation potential for the subsequent cash crop. Further analysis revealed that the abundance of two OTUs, both of which were classified as *A. morrowiae*, were driving the dissimilarity between the 3Spp mixture and the other CCs. In a study addressing plant-fungal inoculum feedback, *Panicum sphaerocarpon* was found to promote the growth of *A. morrowiae*, a fungus that promotes the growth of a second plant, *Plantago lanceolate*^66^. Interestingly, *P. lanceolata* does not promote the growth of *A. morrowiae*. Feedbacks may also be playing a role in the 3Spp mixture that result in the promotion or reduction of certain AM fungal groups including *A. morrowiae*.

Identifying suitable cover crops for a particular context is highly dependent on the goals of the farm manager, and the use of cover crop mixtures can provide multiple ecosystem services in a single growing season^6–9,65^. Not all cover crops will have similar impacts on soil microbes, e.g., some cover crops have been associated with increased AM fungal biomass and hyphal densities^15,36^, while others support increases in non-AM fungi^17,35,36^.

Our findings support two hypotheses put forward by^36^. The first hypothesis, that there are cover crop-specific effects on microbial communities, is supported by the differences in composition of AM fungi and Core OTUs across the CCs. The second hypothesis, that cover crop mixtures maintain certain characteristics of each cover crop species within that mixture, is supported by the similarity of fungal community composition in the 6Spp mixture compared to the monocultures. One interesting finding herein was that the 3Spp mixture had a different fungal composition compared to the 6Spp mixture. One explanation for this would be that ecosystem services provided by cover crop mixtures are dependent on the functional diversity included in the mixture^7,36^. Alternatively, there may be unique plant-fungi feedbacks like those mentioned in^66^ occuring in the 3Spp mixture that support different compositions of fungi compared to the 6Spp mixture or the monocultures. Given the importance of AM fungi as plant growth promoters and the potential importance of cover crop mixtures in supporting different compositions of AM fungi, further research should be conducted to identify mixtures that promote the growth of AM fungi that can colonize the subsequent cash crop.

Cover crop mixtures can be used to provide other ecosystem services apart from increasing AM fungal propagation. Increasing multifunctionality is of interest to researchers and farmers aiming to improve soil quality, pest suppression, nitrogen supply and retention and to provide many other services^8,67,68^. One key hypothesis has been that increases in aboveground diversity will increase belowground diversity^69,70^. However, the results in the present study failed to support this hypothesis, as there were no differences in alpha-diversity among fallow, monocultures, or polyculture treatments. The disconnect between aboveground and belowground diversity in this study may be due to legacy effects from past management practices such as using Cu-based fungicide, intensive tillage and yearly addition of manure, or a combination of these practices. Another possible explanation is that diversity indices derived from DNA are too insensitive to detect short-term microbial responses to aboveground plant diversity^31^. Because the present study was not designed to identify how such practices (i.e., history of fungicide application, tillage, and manure additions) impact fungal communities, future studies would need to incorporate comparisons such as synthetic fertilizer versus manure addition, no-till versus tillage, or fungicide usage to better understand how cover cropping with both monocultures and polycultures impact soil fungi.

## Conclusions

In this experiment, we found that relative abundances of fungal groups depended most strongly on sampling time and that different groups of fungi (i.e., AM fungi compared to Core OTUs) responded differently to CC treatments. Genera associated with AM and Insect Pathogens were impacted by both CC species and CC Function. This suggests that intentional management of CCs may enable farmers to manage for particular goals, like increased mycorrhizal colonization of cash crops, or decreased belowground pest pressure. Additionally, cover crop mixtures (3Spp and 6Spp) can support either distinct or similar fungal communities compared to monocultures, depending on the species mixture. This study highlights both the importance of soil heterogeneity, as well as the importance of cover crop identity and diversity in driving fungal community compositions and potential fungal functions (Insect Pathogens and AM fungi). While this research indicates the immediate effect of CC on fungal communities, increased temporal sampling over several seasons and/or years would need to be performed to determine if CC persistently impacts fungal assemblages. Future research comparing cover crops in both mono- and poly-cultures, combined with multifunctionality measurements and fungal community assessments, can help elucidate cover cropping effects on soil fungi to  improve overall cropping system performance.

## Methods

### Site description and sample collections

Soil samples were collected from an organic transition experiment established in 2012 at the Russell E. Larson Agricultural Research Center near Rock Springs, PA that has been previoulsy described by Murrell *et al*.^9^. The dominant soil series at this site is Murrill channery silt loam (fine-loamy, mixed, semiactive, mesic Typic Hapludult) with 0 to 3% slope. A layout of the plots is shown in Supplementary Fig. 5. Experimental plots (9.1 m x 6.5 m) were maintained in a three-year crop rotation (corn-soybean-wheat), with winter cover crops planted each year following harvest of the main crop. In fall 2014, cover crops were planted after wheat harvest and terminated via flail mowing the following spring. After cover crop termination, dairy bedded-pack manure was incorporated (47 Mg ha^−1^) into the plots via moldboard plowing. Corn was planted on May 28, 2015.

The complete experiment consisted of 12 cover crop treatments, replicated 4 times using a randomized block design, and of these, 9 cover crop treatments (36 plots) were chosen for fungal community analysis. These included six individual species: canola (*Brassica napus* L. cultivar Wichita); common medium red clover (*Trifolium pratense* L.); oat (*Avena sativa* L. cultivar Jerry); Austrian winter pea (*Pisum sativum* L. cultivar Arvika); oilseed radish (*Raphanus sativus* L, cultivar Tillage Radish); and cereal rye (*Secale cereal*e L., cultivar Aroostook). In addition, we included a three-species mixture (3Spp) of Austrian winter pea, common medium red clover, and cereal rye; a six-species mixture (6Spp) of Austrian winter pea, common medium red clover, cereal rye, canola, oat and oilseed radish; and a fallow treatment. Despite good establishment of the mixtures in the fall, Rye was the dominant species in the spring due to winter kill of other species. Weeds were not removed from any plots during the experiment. Seeding rates can be found in Murrell *et al*.^9^.

Soils planted in cover crops during the fall of 2014 were sampled twice in 2015, which represented the fourth year of the experiment. Plots were sampled on 6 May 2015 (before the cover crop was terminated by mowing) and on 16 July 2015 (when plots were in midseason corn), for a total of 72 samples (plots for sampling are denoted in Supplementary Fig. 5). Composite bulk soil samples (ten cores of 3.1 cm diameter at 20 cm depth) were collected from each plot. Composited cores were thoroughly mixed, and portions removed for storage at −80 °C until further analysis.

### Soil chemical and physical measurements and analyses

Data from chemical and physical tests was performed on soils from the same plots and used to relate soil properties to fungal community data. Soil tests conducted by the Penn State Agricultural Analytical Services Laboratory included P, K, Mg, Ca, S, Zn, Cu, CEC, CEC-K%, CEC-CA%, CEC-Mg% and soil organic matter by loss-on-ignition (LOI). Additional tests were pH and electrical conductivity (1:2 soil:water slurries), particle size analysis by the pipet method^71^, gravimetric water content^72^, matric potential^73^, and POXC^74^. Soil organic matter determined by LOI reflects both labile and recalcitrant organic matter in the soil, whereas permanganate oxidizable carbon reflects the labile pool that supports biological activity more readily^75^.

#### DNA extraction, ITS gene sequencing, and analysis

Approximately 0.25 g of rapidly thawed soil from the composited samples were used for DNA extraction with the MoBio PowerSoil DNA isolation kit (MoBio Laboratories, Inc., Carlsbad, CA) following manufacturer’s instructions. Extracts were shipped to Molecular Research DNA Laboratory (MRDNA, www.mrdnalab.com; Shallowater, TX, USA). Library preparation was performed with PCR using the HotStarTaq Master Mix Kit (Qiagen, USA) fungal ITS primers: ITS1-F CTTGGTCATTTAGAGGAAGTAA and ITS2 GCTGCGTTCTTCATCGATGC^76^. Primer pairs included Illumina Nextera adapters, linkers, and barcodes. Amplification conditions were: 94 °C for 3 minutes, followed by 35 cycles of 94 °C for 30 seconds, 53 °C for 40 seconds and 72 °C for 1 minute, with a final elongation step at 72 °C for 5 minutes. Amplified products were checked on a 2% agarose gel using gel electroctrophoresis. Samples were pooled into equal molar concentrations based on molecular weight and DNA concentrations and purified using Ampure XP beads (Beckman Coulter, USA). Libraries were then sequenced by paired-end sequencing (2 × 250 bp) with an Illumina MiSeq sequencer.

Samples were demultiplexed and barcodes and primers were removed from sequences. Sequences less than 150 bp and/or with ambiguous base calls were removed in QIIME (version 1.8.0^77^). Forward and reverse reads were joined and operational taxonomic units (OTUs) were obtained by clustering sequences based on 97% similarity using USEARCH (version 10.0.240^78^). Chimeric sequences were removed with UCHIME in conjunction with QIIME^77,79^. Taxonomic classification of OTUs was carried out with BLASTn and a curated database (MR DNA, Shallowater, TX) derived from RDPII and NCBI with an 80% identify cutoff. Singletons and sequences from non-fungal Eukarya were removed. Rarefactions curves were generated in R Studio (version 3.5.2^80^) using vegan (package version 2.5–6^81^).

Relative abundances of OTUs were calculated and used to create phylum and class-level heatmaps of the top five phyla/classes from each sample, while OTU-level heatmaps were created using the top 20 OTUs across all samples. Samples were rarefied at a depth of 14 737 sequences for alpha-diversity estimates only. Sequence sets were rarefied ten times and repeated subsamples averaged to obtain representative Shannon diversity index and species richness (OTU richness). A linear mixed effect model was created in R using the lme4 (version 1.1–21) and lmerTest packages (version 3.1–0) followed by pairwise comparisons using estimated marginal means (emmeans; version 1.4.1) with Tukey p-value adjustments to determine differences in alpha-diversity among fixed variables using block as the random variable^82–84^. Sets of fixed variables included CCD; CC; Function; season (spring before cover crop termination vs summer after plowing and corn establishment); the interaction between CC*Season (e.g., Pea-spring vs Pea-summer); or the interaction between Function*Season (e.g,, Legume-spring vs Legume-summer).

To facilitate beta-diversity comparisons, OTUs from all 72 samples were sorted into six groupings. Four groupings were based on abundance (All, Rare, Abundant, Core) and two were based on genera with recognized functions (potential Insect Pathogens and AM fungi; Supplementary Table 8). An OTU was defined as “core” if it was found in all 72 samples. Rare OTUs were defined as accounting for less than 0.1% of the fungal sequences in every sample, while abundant OTUs were defined as those accounting for >0.1% of sequences in any one sample.

Genera assigned to the Insect Pathogen or AM fungal group were subjected to linear mixed modeling but only if the genus was observed in at least 50% of the samples. Because one of the assumptions for linear models is normally distributed residuals, transformations were performed on genus abundances when necessary. A separate linear mixed effect model was created for each genus using CC, Function, Season, CC*Season, and Function*Season as fixed effects and block as the random effect with the lme4 package^82^. Adjustments of p-values were performed on lmer outputs using false discovery rate (fdr) corrections^85^. Pairwise comparisons for each statistically significant model were performed with Tukey p-value adjustments using the emmeans package^84^.

To test the effect of cover crops on fungal community composition, each fungal OTU grouping was subjected to global PERMANOVA tests in R using vegan to determine whether CC, Function, Season or the interaction of CC*Season or Function* Season impacted fungal assemblages with 999 permutations. Pairwise comparisons were performed using PERMANOVA with Bray-Curtis distances and fdr p-value adjustments with the RVAideMemoire package (version 0.9–73) if the global PERMANOVA resulted in a p-value <0.05^86^. Relational diagrams were constructed to show pairwise treatment groups that had different community compositions (p < 0.05). Further analysis was performed to identify OTUs that were driving the dissimilarities observed between the 3Spp mixture compared to the other CC’s using the simper function in the vegan package.

### Analysis with community composition and environmental data

Detrended correspondence analyses were performed with vegan for each OTU grouping to assess axes lengths, which were all>4 except for Rare, which had a length of 3.3. Thus, canonical correspondence analyses (CCA) models were built for each OTU grouping using the vegan in R. Forward and backward selection was performed with Ordistep to determine the best fitting model (lowest AIC) for explaining the dispersion of samples within the fungal groupings. Environmental variables that were identified from ordistep were used as explanatory vectors in canonical correspondence analysis (CCA) plots. ANOVAs were then performed on the CCA models to determine whether the model could explain a significant portion of the inertia. Variance inflation factors were assessed for each model using the vif.cca function from the vegan package.

## Supplementary information

supplementary information.

## Data Availability

Sequences were deposited in the National Center for Biotechnology Information under Bioproject ID: PRJNA481943. Taxonomic information, as well as relative abundance data, for each OTU assigned to the Core group, and untransformed environmental data can be found here https://github.com/maracashay/Cover-Crop-Fungal-Communities.
