# Supplementary material for: Fungal community shifts in soils with varied cover crop treatments and edaphic properties
Source: Sci Rep. 2020 Apr 10;10:6198. doi: 10.1038/s41598-020-63173-7 (PMC7148350; doi:10.1038/s41598-020-63173-7)
Supplement: Supplementary file 1 — supplementary information. [file 41598_2020_63173_MOESM1_ESM.docx]

**Fungal community shifts in soils with varied cover crop treatments and edaphic properties**

Mara L. Cloutier^1,2*^, Ebony Murrell^3^, Mary Barbercheck^4^, Jason Kaye^1,2^, Denise Finney^5^, Irene García-González^6^, and Mary Ann Bruns^1,2^

^1^Department of Ecosystem Science and Management and ^2^Biogeochemistry Dual Title PhD Program--Pennsylvania State University, University Park, 16801, PA, USA

^3^The Land Institute, Salina, 67401, KS, USA

^4^Department of Entomology-Pennsylvania State University, University Park, 16801, PA, USA

^5^Department of Biology, Ursinus College, Collegeville, 19426, PA, USA

^6^Departamento de Producción Agraria, Universidad Politécnica de Madrid, Avda. Complutense s/n, 28040 Madrid, Spain

Mara Cloutier [muc345@psu.edu](mailto:muc345@psu.edu)

Ebony Murrell [murrell@landinstitute.org](mailto:murrell@landinstitute.org)

Mary Barbercheck [meb34@psu.edu](mailto:meb34@psu.edu)

Jason Kaye [jpk12@psu.edu](mailto:jpk12@psu.edu)

Denise Finney [dfinney@ursinus.edu](mailto:dfinney@ursinus.edu)

Irene García-González [irene.gargon@gmail.com](mailto:irene.gargon@gmail.com)

^*^Corresponding Author, [muc345@psu.edu](mailto:muc345@psu.edu)

Figure Supplements


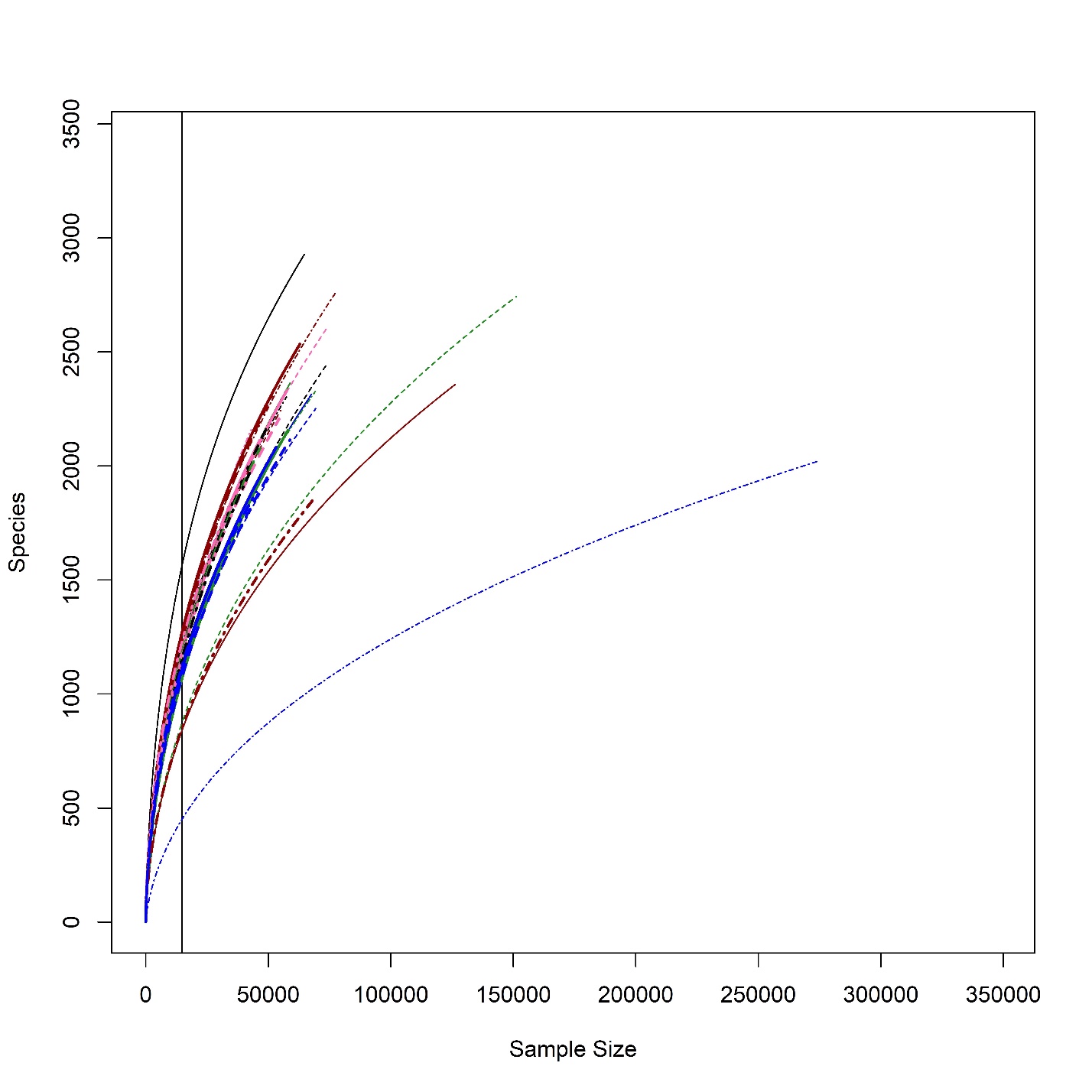


Supplementary Figure 1. Rarefaction curve of fungal OTUs across the 72 samples.


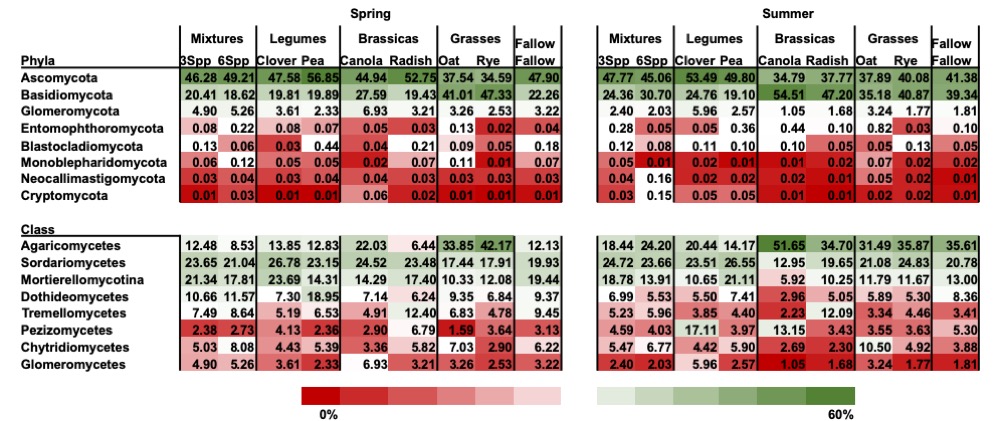


Supplementary Figure 2. Phyla and Class-level relative abundances across the different cover crop treatments and plant functions in the spring and summer samples.


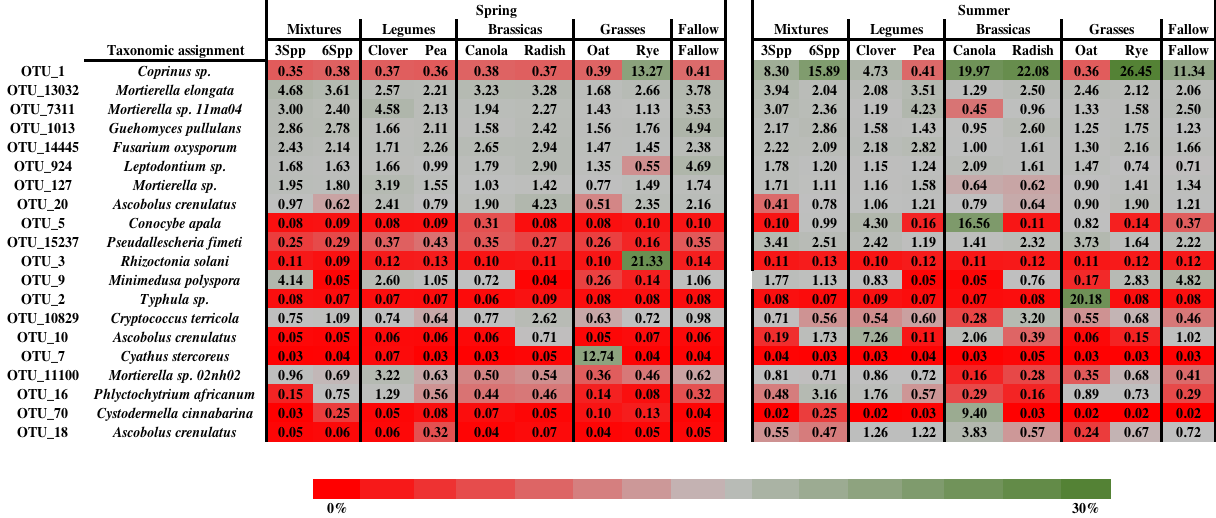


Supplementary Figure 3. Heatmap showing the relative abundances of the top 20 OTUs from the cover crop treatments in spring and summer.


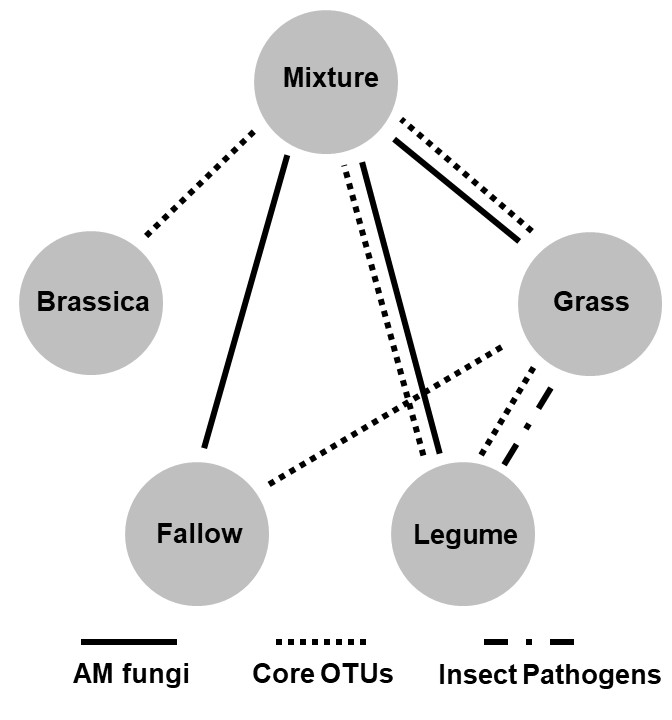
Supplemental Figure 4. Relational diagram showing differences (p-value <0.05) among CC Function calculated by pairwise PERMANOVA comparisons using the Bray-Curtis dissimilarity matrix (n=8 for Fallow, n=16 for other CC Functions).


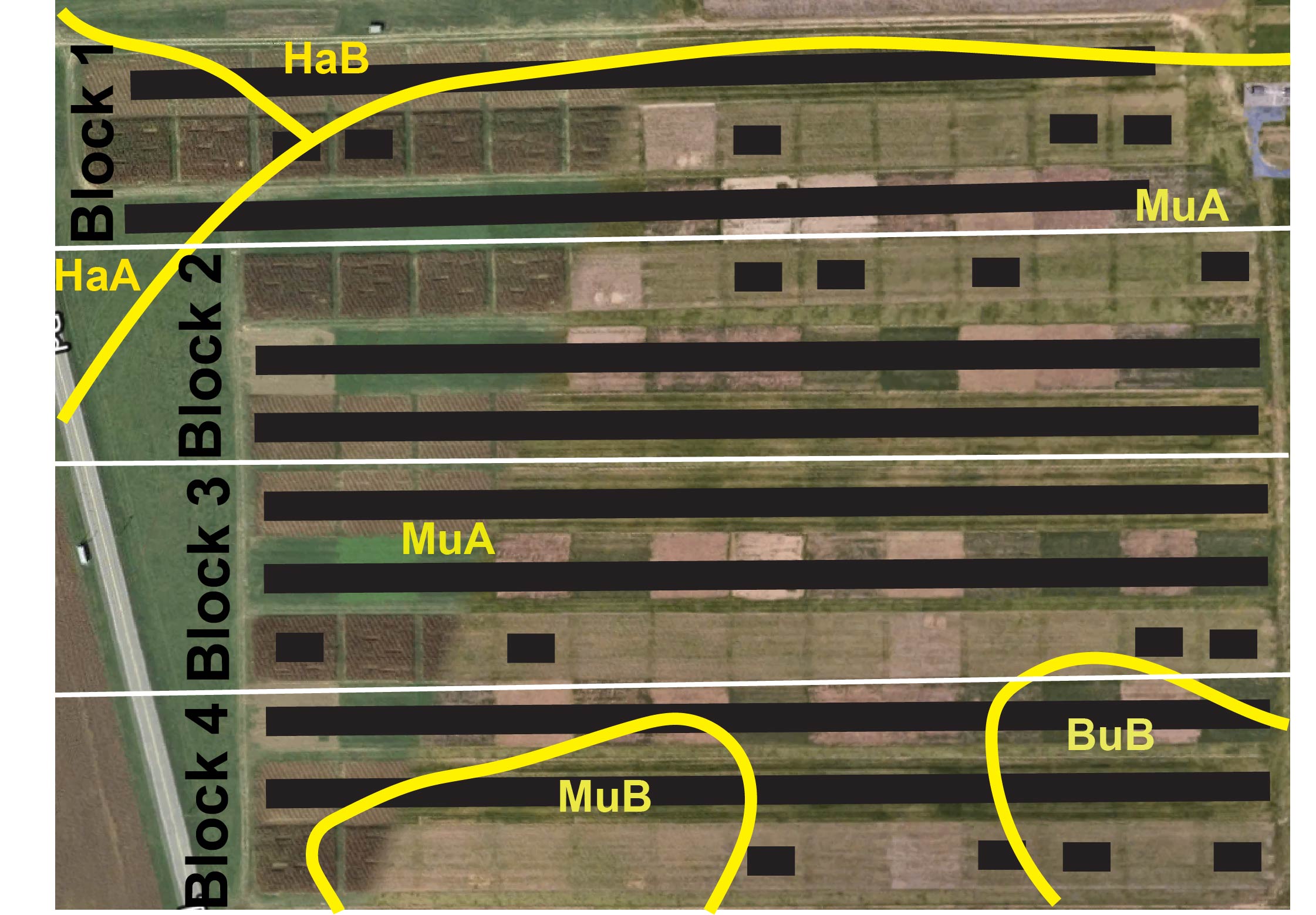


Supplementary Figure 5. Plot map showing the complete randomized block design. Four soil mapping units are found across the experimental site: Murrill channery silt loam on a 0-3 % slope (MuA), Murrill channery silt loam on a 3-8 % slope (MuB), Hagerstown silt loam on a 3-8 % slope (HaB), and Buchanan-Andover gravelly loam on a 3-8 % slop (BuB). Because the experiment comprises a full-entry, three-year rotation, each crop in the corn-soybean-wheat rotation is present every year. In the present study, plots soybean and wheat were not sampled, and they are shown as being blacked out in the figure above. Some plots in corn that were not among this study’s nine treatments, are also blacked out. Image was taken with Google Satellite imagery (<https://www.google.com/maps/@40.7206546,-77.9265365,586a,35y,340.61h,7.49t/data=!3m1!1e3>). Soil mapping units were overlayed from the Web Soil Survey (<https://websoilsurvey.sc.egov.usda.gov/App/WebSoilSurvey.aspx>).

Supplementary Table 1. Number of sequences, total number of bases and average length of the sequences per sample. First number after the CC species is the field block and the second number represents whether the sample was taken in the spring (1) or summer (2).

| **Sample** | **Sequences** | **Bases** | **Average Length** |
| --- | --- | --- | --- |
| 3Spp-1-1 | 64388 | 19452272 | 280.1 |
| 3Spp-1-2 | 78353 | 23697369 | 281.7 |
| 3Spp-2-1 | 76523 | 23726914 | 288.1 |
| 3Spp-2-2 | 81632 | 25763145 | 293.6 |
| 3Spp-3-1 | 52106 | 15561889 | 276.7 |
| 3Spp-3-2 | 155619 | 48437011 | 289.3 |
| 3Spp-4-1 | 41474 | 12543510 | 280.4 |
| 3Spp-4-2 | 52660 | 15707442 | 276.3 |
| 6Spp-1-1 | 69145 | 21290188 | 285.9 |
| 6Spp-1-2 | 17972 | 5602048 | 289.7 |
| 6spp-2-1 | 78090 | 25143836 | 300 |
| 6spp-2-2 | 70171 | 21009995 | 277.4 |
| 6spp-3-1 | 57398 | 17214708 | 277.9 |
| 6spp-3-2 | 195495 | 65019103 | 310.6 |
| 6Spp-4-1 | 62081 | 21400492 | 322.7 |
| 6Spp-4-2 | 92528 | 29145397 | 293 |
| Canola-1-1 | 56226 | 17888044 | 296.1 |
| Canola-1-2 | 44776 | 13740658 | 284.9 |
| Canola-2-1 | 56965 | 18126044 | 296.2 |
| Canola-2-2 | 224166 | 76356961 | 318.6 |
| Canola-3-1 | 75628 | 23873782 | 293.7 |
| Canola-3-2 | 71284 | 22495048 | 293.6 |
| Canola-4-1 | 47706 | 14702696 | 286.2 |
| Canola-4-2 | 92696 | 30822657 | 310.5 |
| Clover-1-1 | 88803 | 29179924 | 306.6 |
| Clover-1-2 | 86725 | 27147994 | 291 |
| Clover-2-1 | 63783 | 18638512 | 270.2 |
| Clover-2-2 | 93836 | 29498832 | 292.4 |
| Clover-3-1 | 62803 | 19109638 | 282.3 |
| Clover-3-2 | 191011 | 63685761 | 311.4 |
| Clover-4-1 | 51557 | 16119473 | 290.7 |
| Clover-4-2 | 92583 | 28821382 | 289.3 |
| Fallow-1-1 | 86706 | 26281857 | 281.1 |
| Fallow-1-2 | 75126 | 22806445 | 281.6 |
| Fallow-2-1 | 49521 | 14805869 | 277 |
| Fallow-2-2 | 141616 | 46860907 | 308.9 |
| Fallow-3-1 | 67075 | 21117522 | 292.8 |
| Fallow-3-2 | 54048 | 16824858 | 289.3 |
| Fallow-4-1 | 44821 | 13817648 | 286.3 |
| Fallow-4-2 | 93130 | 29691099 | 296.8 |
| Oat-1-1 | 171134 | 60001073 | 328.6 |
| Oat-1-2 | 76592 | 23299902 | 282.2 |
| Oat-2-1 | 97087 | 30735623 | 294.6 |
| Oat-2-2 | 96043 | 30388741 | 294.4 |
| Oat-3-1 | 73704 | 23392344 | 295.4 |
| Oat-3-2 | 362988 | 125792833 | 324.5 |
| Oat-4-1 | 102176 | 32247438 | 293.6 |
| Oat-4-2 | 100442 | 33114152 | 307.7 |
| Pea-1-1 | 81574 | 2475694 | 281.6 |
| Pea-1-2 | 61507 | 18315371 | 275.8 |
| Pea-2-1 | 75938 | 23078653 | 281.9 |
| Pea-2-2 | 163825 | 50733965 | 287.7 |
| Pea-3-1 | 63091 | 19246135 | 283.1 |
| Pea-3-2 | 121988 | 38199189 | 291.1 |
| Pea-4-1 | 77940 | 24888402 | 297.3 |
| Pea-4-2 | 74624 | 22799373 | 283.5 |
| Radish-1-1 | 76634 | 23674384 | 286.9 |
| Radish-1-2 | 90250 | 29144007 | 300.9 |
| Radish-2-1 | 84191 | 26050585 | 287.4 |
| Radish-2-2 | 79073 | 24083045 | 282.6 |
| Radish-3-1 | 59937 | 18021163 | 278.7 |
| Radish-3-2 | 118497 | 36814646 | 288.7 |
| Radish-4-1 | 44796 | 14037756 | 291.4 |
| Radish-4-2 | 124731 | 40214036 | 300.4 |
| Rye-1-1 | 138065 | 46015831 | 311.3 |
| Rye-1-2 | 25354 | 7281709 | 277 |
| Rye-2-1 | 281417 | 92637936 | 307.2 |
| Rye-2-2 | 76060 | 23475975 | 286.7 |
| Rye-3-1 | 48106 | 14181476 | 272.8 |
| Rye-3-2 | 127570 | 40749243 | 297.4 |
| Rye-4-1 | 59848 | 19342255 | 301.2 |
| Rye-4-2 | 199880 | 67192389 | 314.2 |

Supplementary Table 2. Absolute (Abs) and relative (Rel) abundances of OTU groupings across the 72 samples. All relative abundances were calculated by dividing the absolute abundances by the total number of sequences per sample.

| **Sample** | **AM fungi** | | **Insect Pathogens** | | **Abundant** | | **Core** | | **Rare** | | **Total # of Seqs** |
| --- | --- | --- | --- | --- | --- | --- | --- | --- | --- | --- | --- |
|  | **Abs** | **Rel** | **Abs** | **Rel** | **Abs** | **Rel** | **Abs** | **Rel** | **Abs** | **Rel** |  |
| 3Spp-1-1 | 1244 | 0.029 | 94 | 0.002 | 11781 | 0.921 | 52293 | 0.756 | 5393 | 0.078 | 69200 |
| 3Spp-1-2 | 986 | 0.023 | 82 | 0.002 | 39896 | 0.929 | 35190 | 0.819 | 2958 | 0.069 | 42968 |
| 3Spp-2-1 | 3162 | 0.055 | 243 | 0.004 | 54035 | 0.940 | 43654 | 0.759 | 3414 | 0.059 | 57512 |
| 3Spp-2-2 | 1208 | 0.023 | 62 | 0.001 | 49960 | 0.937 | 34306 | 0.644 | 3232 | 0.061 | 53294 |
| 3Spp-3-1 | 2060 | 0.029 | 72 | 0.001 | 66705 | 0.933 | 59523 | 0.833 | 4592 | 0.064 | 71479 |
| 3Spp-3-2 | 1024 | 0.014 | 92 | 0.001 | 68256 | 0.947 | 62884 | 0.873 | 3659 | 0.051 | 72059 |
| 3Spp-4-1 | 29190 | 0.084 | 329 | 0.001 | 323831 | 0.928 | 251139 | 0.720 | 24509 | 0.070 | 348869 |
| 3Spp-4-2 | 6469 | 0.036 | 350 | 0.002 | 162335 | 0.913 | 139392 | 0.784 | 14953 | 0.084 | 177819 |
| 6Spp-1-1 | 3842 | 0.057 | 124 | 0.002 | 62729 | 0.927 | 51348 | 0.759 | 5627 | 0.083 | 67652 |
| 6Spp-1-2 | 1732 | 0.023 | 111 | 0.001 | 69518 | 0.934 | 61420 | 0.826 | 4893 | 0.066 | 74394 |
| 6spp-2-1 | 2322 | 0.047 | 41 | 0.001 | 45804 | 0.925 | 36020 | 0.727 | 3661 | 0.074 | 49530 |
| 6spp-2-2 | 1390 | 0.027 | 104 | 0.002 | 47363 | 0.924 | 38214 | 0.745 | 3779 | 0.074 | 51282 |
| 6spp-3-1 | 3520 | 0.042 | 191 | 0.002 | 77408 | 0.933 | 64191 | 0.774 | 5435 | 0.065 | 82982 |
| 6spp-3-2 | 403 | 0.006 | 26 | 0.000 | 64458 | 0.974 | 61186 | 0.924 | 1731 | 0.026 | 66208 |
| 6Spp-4-1 | 12157 | 0.065 | 153 | 0.001 | 172121 | 0.915 | 127254 | 0.676 | 15743 | 0.084 | 188192 |
| 6Spp-4-2 | 3026 | 0.027 | 135 | 0.001 | 101828 | 0.916 | 83009 | 0.747 | 9139 | 0.082 | 111126 |
| Canola-1-1 | 4447 | 0.081 | 126 | 0.002 | 51056 | 0.927 | 37213 | 0.676 | 4101 | 0.074 | 55068 |
| Canola-1-2 | 2199 | 0.015 | 47 | 0.000 | 146702 | 0.970 | 133268 | 0.881 | 4655 | 0.031 | 151301 |
| Canola-2-1 | 6004 | 0.155 | 36 | 0.001 | 35865 | 0.924 | 29426 | 0.758 | 2874 | 0.074 | 38821 |
| Canola-2-2 | 283 | 0.006 | 17 | 0.000 | 43186 | 0.975 | 37642 | 0.850 | 1084 | 0.024 | 44309 |
| Canola-3-1 | 1345 | 0.010 | 149 | 0.001 | 129496 | 0.959 | 100263 | 0.742 | 5187 | 0.038 | 135055 |
| Canola-3-2 | 549 | 0.007 | 46 | 0.001 | 72431 | 0.958 | 61243 | 0.810 | 3159 | 0.042 | 75593 |
| Canola-4-1 | 1584 | 0.032 | 147 | 0.003 | 45171 | 0.918 | 37869 | 0.769 | 3937 | 0.080 | 49223 |
| Canola-4-2 | 1732 | 0.015 | 89 | 0.001 | 113027 | 0.951 | 102550 | 0.863 | 5654 | 0.048 | 118806 |
| Clover-1-1 | 1668 | 0.027 | 230 | 0.004 | 58177 | 0.925 | 40199 | 0.639 | 5910 | 0.094 | 62919 |
| Clover-1-2 | 1035 | 0.018 | 89 | 0.002 | 54739 | 0.943 | 40703 | 0.701 | 3155 | 0.054 | 58038 |
| Clover-2-1 | 1372 | 0.036 | 83 | 0.002 | 36272 | 0.940 | 32512 | 0.843 | 2191 | 0.057 | 38569 |
| Clover-2-2 | 1597 | 0.024 | 271 | 0.004 | 62052 | 0.932 | 55641 | 0.836 | 4401 | 0.066 | 66576 |
| Clover-3-1 | 369 | 0.025 | 30 | 0.002 | 13764 | 0.934 | 9785 | 0.664 | 934 | 0.063 | 14737 |
| Clover-3-2 | 442 | 0.010 | 62 | 0.001 | 40444 | 0.958 | 37753 | 0.894 | 1739 | 0.041 | 42236 |
| Clover-4-1 | 8232 | 0.058 | 222 | 0.002 | 132768 | 0.929 | 118853 | 0.832 | 9965 | 0.070 | 142870 |
| Clover-4-2 | 29181 | 0.266 | 315 | 0.003 | 103672 | 0.945 | 92072 | 0.840 | 5897 | 0.054 | 109654 |
| Fallow-1-1 | 1318 | 0.023 | 84 | 0.001 | 53700 | 0.935 | 48714 | 0.849 | 3598 | 0.063 | 57410 |
| Fallow-1-2 | 1856 | 0.024 | 146 | 0.002 | 73308 | 0.942 | 57466 | 0.738 | 4411 | 0.057 | 77852 |
| Fallow-2-1 | 1443 | 0.021 | 124 | 0.002 | 62824 | 0.921 | 54823 | 0.804 | 5170 | 0.076 | 68210 |
| Fallow-2-2 | 210 | 0.006 | 17 | 0.000 | 32910 | 0.969 | 29340 | 0.864 | 1038 | 0.031 | 33959 |
| Fallow-3-1 | 1295 | 0.019 | 126 | 0.002 | 64145 | 0.938 | 42519 | 0.622 | 4026 | 0.059 | 68366 |
| Fallow-3-2 | 3111 | 0.023 | 259 | 0.002 | 128093 | 0.945 | 83641 | 0.617 | 7081 | 0.052 | 135563 |
| Fallow-4-1 | 7482 | 0.066 | 200 | 0.002 | 106173 | 0.934 | 96317 | 0.848 | 7217 | 0.064 | 113642 |
| Fallow-4-2 | 1624 | 0.020 | 117 | 0.001 | 78271 | 0.951 | 71214 | 0.865 | 3830 | 0.047 | 82331 |
| Oat-1-1 | 4287 | 0.016 | 245 | 0.001 | 265371 | 0.965 | 243094 | 0.884 | 9480 | 0.034 | 274938 |
| Oat-1-2 | 1390 | 0.030 | 124 | 0.003 | 42102 | 0.922 | 35569 | 0.779 | 3504 | 0.077 | 45649 |
| Oat-2-1 | 2333 | 0.050 | 88 | 0.002 | 43020 | 0.922 | 35282 | 0.756 | 3610 | 0.077 | 46668 |
| Oat-2-2 | 4061 | 0.075 | 32 | 0.001 | 52015 | 0.956 | 42841 | 0.788 | 2350 | 0.043 | 54386 |
| Oat-3-1 | 2751 | 0.053 | 92 | 0.002 | 48335 | 0.930 | 41779 | 0.804 | 3545 | 0.068 | 51960 |
| Oat-3-2 | 78 | 0.004 | 12 | 0.001 | 21497 | 0.982 | 20725 | 0.946 | 582 | 0.027 | 21897 |
| Oat-4-1 | 1024 | 0.012 | 59 | 0.001 | 81100 | 0.966 | 63692 | 0.758 | 2828 | 0.034 | 83981 |
| Oat-4-2 | 4010 | 0.021 | 241 | 0.001 | 176070 | 0.923 | 157406 | 0.825 | 14242 | 0.075 | 190824 |
| Pea-1-1 | 2532 | 0.039 | 107 | 0.002 | 55897 | 0.863 | 46904 | 0.725 | 8742 | 0.135 | 64738 |
| Pea-1-2 | 2776 | 0.022 | 493 | 0.004 | 114678 | 0.908 | 102284 | 0.810 | 11142 | 0.088 | 126231 |
| Pea-2-1 | 748 | 0.021 | 98 | 0.003 | 32870 | 0.914 | 27480 | 0.764 | 3042 | 0.085 | 35976 |
| Pea-2-2 | 1035 | 0.011 | 363 | 0.004 | 83031 | 0.913 | 71733 | 0.789 | 7615 | 0.084 | 90912 |
| Pea-3-1 | 1032 | 0.017 | 89 | 0.001 | 56132 | 0.930 | 45025 | 0.746 | 4055 | 0.067 | 60377 |
| Pea-3-2 | 1082 | 0.013 | 117 | 0.001 | 79571 | 0.946 | 67836 | 0.806 | 4298 | 0.051 | 84119 |
| Pea-4-1 | 1415 | 0.016 | 118 | 0.001 | 81634 | 0.949 | 39371 | 0.458 | 4316 | 0.050 | 86048 |
| Pea-4-2 | 3695 | 0.057 | 190 | 0.003 | 60520 | 0.932 | 50913 | 0.784 | 4258 | 0.066 | 64907 |
| Radish-1-1 | 1297 | 0.017 | 136 | 0.002 | 70099 | 0.940 | 56340 | 0.755 | 4353 | 0.058 | 74582 |
| Radish-1-2 | 1445 | 0.021 | 29 | 0.000 | 66728 | 0.962 | 63171 | 0.911 | 2584 | 0.037 | 69360 |
| Radish-2-1 | 3206 | 0.054 | 91 | 0.002 | 54352 | 0.924 | 49249 | 0.837 | 4386 | 0.075 | 58839 |
| Radish-2-2 | 1147 | 0.027 | 43 | 0.001 | 39567 | 0.934 | 31211 | 0.736 | 2733 | 0.064 | 42385 |
| Radish-3-1 | 1523 | 0.022 | 80 | 0.001 | 63328 | 0.926 | 54681 | 0.799 | 4819 | 0.070 | 68401 |
| Radish-3-2 | 594 | 0.009 | 59 | 0.001 | 60421 | 0.955 | 57088 | 0.903 | 2694 | 0.043 | 63250 |
| Radish-4-1 | 2721 | 0.035 | 123 | 0.002 | 71975 | 0.928 | 64730 | 0.834 | 5417 | 0.070 | 77575 |
| Radish-4-2 | 843 | 0.010 | 85 | 0.001 | 80638 | 0.955 | 71273 | 0.844 | 3680 | 0.044 | 84438 |
| Rye-1-1 | 967 | 0.016 | 31 | 0.001 | 56797 | 0.965 | 52364 | 0.889 | 2164 | 0.037 | 58879 |
| Rye-1-2 | 783 | 0.017 | 80 | 0.002 | 43716 | 0.944 | 32931 | 0.711 | 2577 | 0.056 | 46327 |
| Rye-2-1 | 198 | 0.005 | 33 | 0.001 | 42549 | 0.989 | 41171 | 0.957 | 457 | 0.011 | 43007 |
| Rye-2-2 | 1021 | 0.023 | 57 | 0.001 | 41981 | 0.929 | 35149 | 0.778 | 3050 | 0.068 | 45174 |
| Rye-3-1 | 2085 | 0.032 | 142 | 0.002 | 60119 | 0.932 | 53609 | 0.831 | 4090 | 0.063 | 64508 |
| Rye-3-2 | 4569 | 0.022 | 699 | 0.003 | 200512 | 0.952 | 175829 | 0.835 | 9714 | 0.046 | 210529 |
| Rye-4-1 | 3249 | 0.048 | 248 | 0.004 | 61798 | 0.912 | 49126 | 0.725 | 5859 | 0.087 | 67727 |
| Rye-4-2 | 447 | 0.010 | 34 | 0.001 | 44499 | 0.972 | 41534 | 0.907 | 1254 | 0.027 | 45789 |

Supplementary Table 3. Measurements of evenness and richness using Shannon diversity index and species richness (total OTU count). Values here are averages for the cover crops (CC) across the four blocks for the spring and summer samples (n=4). Values in () correspond to standard errors.

| **Season** | **CC** | **Shannon** | **Richness** |
| --- | --- | --- | --- |
| **Spring** | **3Spp** | 5.04 (±0.08) | 2099 (±100) |
|  | **6Spp** | 5.25 (±0.07) | 2218 (±74) |
|  | **Canola** | 4.85 (±0.29) | 1985 (±61) |
|  | **Radish** | 5.06 (±0.09) | 2187 (±129) |
|  | **Clover** | 4.70 (±0.11) | 2186 (±154) |
|  | **Pea** | 5.12 (±0.27) | 2436 (±190) |
|  | **Oat** | 4.27 (±0.58) | 2463 (±180) |
|  | **Rye** | 3.61 (±0.97) | 2153 (±93) |
|  | **Fallow** | 4.86 (±0.17) | 2138 (±129) |
|  |  |  |  |
| **Summer** | **3Spp** | 4.90 (±0.29) | 2627 (±194) |
|  | **6Spp** | 4.41 (±0.66) | 2139 (±355) |
|  | **Canola** | 3.20 (±0.23) | 1881 (±299) |
|  | **Radish** | 4.17 (±0.35) | 2454 (±138) |
|  | **Clover** | 4.33 (±0.25) | 2528 (±180) |
|  | **Pea** | 4.96 (±0.24) | 2703 (±243) |
|  | **Oat** | 3.97 (±0.86) | 2469 (±106) |
|  | **Rye** | 3.98 (±0.64) | 2234 (±334) |
|  | **Fallow** | 4.19 (±0.33) | 2156 (±130) |

Supplementary Table 4. Results from generalized linear mixed modeling with alpha-diversity metrics, species richness and Shannon diversity.

|  |  | **DF** | **Sum of Sqs** | **Mean Sqs** | **F-value** | **Pr(<F)** |
| --- | --- | --- | --- | --- | --- | --- |
| **Richness** | **Function** | 4 | 105150 | 26288 | 0.171 | 0.953 |
|  | **Season** | 1 | 300608 | 300608 | 1.954 | 0.167 |
|  | **Function* Season** | 4 | 217425 | 54356 | 0.353 | 0.841 |
|  |  |  |  |  |  |  |
|  | **CC** | 8 | 429557 | 53695 | 0.373 | 0.930 |
|  | **Season** | 1 | 390286 | 390286 | 2.711 | 0.106 |
|  | **CC* Season** | 8 | 735523 | 91940 | 0.639 | 0.742 |
|  |  |  |  |  |  |  |
|  | **CCD** | 3 | 244253 | 81418 | 0.514 | 0.674 |
|  | **Season** | 1 | 235203 | 235203 | 1.487 | 0.227 |
|  | **CCD* Season** | 3 | 426839 | 142280 | 0.899 | 0.447 |
|  |  |  |  |  |  |  |
| **Shannon** | **Function** | 4 | 4.7211 | 1.1803 | 1.552 | 0.199 |
|  | **Season** | 1 | 4.6961 | 4.6961 | 6.173 | 0.016* |
|  | **Function* Season** | 4 | 3.8102 | 0.9525 | 1.252 | 0.299 |
|  |  |  |  |  |  |  |
|  | **CC** | 8 | 5.8995 | 0.7374 | 0.933 | 0.498 |
|  | **Season** | 1 | 4.7950 | 0.7950 | 6.064 | 0.017* |
|  | **CC* Season** | 8 | 5.3732 | 0.6717 | 0.849 | 0.564 |
|  |  |  |  |  |  |  |
|  | **CCD** | 3 | 0.7312 | 0.2438 | 0.279 | 0.840 |
|  | **Season** | 1 | 2.8992 | 2.8992 | 3.321 | 0.073 |
|  | **CCD* Season** | 3 | 0.5456 | 0.1819 | 0.208 | 0.890 |
| Significant p-values are denoted with an *. | | | | | | |

Supplementary Table 5. Results from PERMANOVA using Bray-Curtis dissimilarity for each OTU grouping by Function and Season.

| **OTU Group** | **Factor** | **DF** | **Sum of Sqs** | **Mean Sqs** | **F.model** | **R^2^** | **Pr(>F)** |
| --- | --- | --- | --- | --- | --- | --- | --- |
| **AM fungi** | **Function** | 4 | 1.377 | 0.344 | 1.590 | 0.083 | 0.006* |
|  | **Season** | 1 | 0.975 | 0.975 | 4.505 | 0.059 | 0.001* |
|  | **Function* Season** | 4 | 0.909 | 0.227 | 1.050 | 0.055 | 0.328 |
|  | **Residuals** | 62 | 16.676 | 0.216 |  |  |  |
|  |  |  |  |  |  |  |  |
| **Insect Pathogens** | **Function** | 4 | 0.803 | 0.201 | 1.575 | 0.084 | 0.043* |
|  | **Season** | 1 | 0.303 | 0.303 | 2.376 | 0.032 | 0.032* |
|  | **Function* Season** | 4 | 0.510 | 0.127 | 0.100 | 0.054 | 0.472 |
|  | **Residuals** | 62 | 7.901 |  |  |  |  |
|  |  |  |  |  |  |  |  |
| **Abundant** | **Function** | 4 | 0.703 | 0.176 | 0.867 | 0.048 | 0.807 |
|  | **Season** | 1 | 0.511 | 0.511 | 2.521 | 0.035 | 0.003* |
|  | **Function* Season** | 4 | 0.748 | 0.187 | 0.923 | 0.052 | 0.660 |
|  | **Residuals** | 62 | 12.563 |  |  |  |  |
|  |  |  |  |  |  |  |  |
| **Rare** | **Function** | 4 | 1.378 | 0.345 | 1.237 | 0.067 | 0.002* |
|  | **Season** | 1 | 0.769 | 0.769 | 2.763 | 0.038 | 0.001* |
|  | **Function* Season** | 4 | 1.065 | 0.266 | 0.957 | 0.052 | 0.757 |
|  | **Residuals** | 62 | 17.262 |  |  |  |  |
|  |  |  |  |  |  |  |  |
| **Core** | **Function** | 4 | 1.012 | 0.253 | 1.533 | 0.079 | 0.013* |
|  | **Time** | 1 | 0.908 | 0.908 | 5.501 | 0.071 | 0.001* |
|  | **Function* Season** | 4 | 0.714 | 0.179 | 1.081 | 0.056 | 0.286 |
|  | **Residuals** | 62 | 10.234 |  |  |  |  |
|  |  |  |  |  |  |  |  |
| **All** | **Function** | 4 | 1.110 | 0.278 | 1.431 | 0.0747 | 0.019* |
|  | **Time** | 1 | 0.905 | 0.905 | 4.666 | 0.0609 | 0.001* |
|  | **Function* Season** | 4 | 0.830 | 0.208 | 1.070 | 0.0558 | 0.285 |
|  | **Residuals** | 62 | 12.024 |  |  |  |  |
| Significant p-values are denoted with an *. | | | | | | | |

Supplementary Table 6. The five most important OTUs driving dissimilarities within the core OTU group between Radish or Rye and 3Spp, 6Spp, Canola, Clover, and Fallow. Individual OTU contributions to total percent dissimilarities between the CCs and genus-level assignments are listed.

|  |  |  | **3Spp** | **6Spp** | **Canola** | **Clover** | **Fallow** |
| --- | --- | --- | --- | --- | --- | --- | --- |
| **Radish** | **1** | **OTU** | 1 | 1 | 1 | 1 | 1 |
|  |  | **ID** | *Coprinus* | *Coprinus* | *Coprinus* | *Coprinus* | *Coprinus* |
|  |  | **%** | 14.9 | 18.4 | 15.7 | 12.6 | 14.7 |
|  | **2** | **OTU** | 9 | 1013 | 5 | 10 | 12 |
|  |  | **ID** | *Minimedusa* | *Guehomyces* | *Conocybe* | *Stachybotrys* | *Psathyrella* |
|  |  | **%** | 3.7 | 3.5 | 8.4 | 4.3 | 4.9 |
|  | **3** | **OTU** | 20 | 20 | 70 | 20 | 9 |
|  |  | **ID** | *Ascobolus* | *Ascobolus* | *Cystodermella* | *Ascobolus* | *Minimedusa* |
|  |  | **%** | 3.0 | 3.0 | 4.7 | 3.1 | 3.6 |
|  | **4** | **OTU** | 10829 | 10829 | 36 | 23 | 1013 |
|  |  | **ID** | *Cryptococcus* | *Cryptococcus* | *Waitea* | *Conocybe* | *Guehomyces* |
|  |  | **%** | 2.8 | 2.7 | 2.8 | 2.9 | 3.5 |
|  | **5** | **OTU** | 48 | 16 | 10829 | 10829 | 924 |
|  |  | **ID** | *Lindtneria* | *Phlyctochytrium* | *Cryptococcus* | *Cryptococcus* | *Leptodontium* |
|  |  | **%** | 2.5 | 2.5 | 2.6 | 2.7 | 3.1 |
| **Rye** | **1** | **OTU** | 1 | 1 | 1 | 1 | 1 |
|  |  | **ID** | *Coprinus* | *Coprinus* | *Coprinus* | *Coprinus* | *Coprinus* |
|  |  | **%** | 22.2 | 23.6 | 21.1 | 19.9 | 21.7 |
|  | **2** | **OTU** | 3 | 3 | 3 | 3 | 3 |
|  |  | **ID** | *Rhizoctonia* | *Rhizoctonia* | *Rhizoctonia* | *Rhizoctonia* | *Rhizoctonia* |
|  |  | **%** | 10.6 | 10.4 | 9.3 | 9.7 | 10.2 |
|  | **3** | **OTU** | 9 | 1013 | 5 | 10 | 12 |
|  |  | **ID** | *Minimedusa* | *Guehomyces* | *Conocybe* | *Stachybotrys* | *Psathyrella* |
|  |  | **%** | 3.8 | 3.3 | 7.9 | 3.6 | 4.4 |
|  | **4** | **OTU** | 1013 | 20 | 70 | 20 | 9 |
|  |  | **ID** | *Guehomyces* | *Ascobolus* | *Cystodermella* | *Ascobolus* | *Minimedusa* |
|  |  | **%** | 2.7 | 2.4 | 4.4 | 2.8 | 3.9 |
|  | **5** | **OTU** | 13032 | 16 | 36 | 9 | 1013 |
|  |  | **ID** | *Mortierella* | *Phlyctochytrium* | *Waitea* | *Minimedusa* | *Guehomyces* |
|  |  | **%** | 2.6 | 2.2 | 2.7 | 2.7 | 3.5 |

Supplementary Table 7. The top five OTUs driving dissimilarities within the AM fungal group

between the 3Spp mixture and other CCs. Individual OTU contributions to total percent

dissimilarities between 3Spp and other CC’s are listed (%).

|  |  | **6Spp** | **Canola** | **Clover** | **Fallow** | **Pea** | **Radish** | **Rye** |
| --- | --- | --- | --- | --- | --- | --- | --- | --- |
| **1** | **OTU** | 154 | 154 | 154 | 154 | 154 | 154 | 154 |
|  | **ID** | Acaulospora | Acaulospora | Acaulospora | Acaulospora | Acaulospora | Acaulospora | Acaulospora |
|  | **%** | 14.0% | 13.2% | 12.4% | 15.4% | 15.2% | 14.4% | 16.8% |
| **2** | **OTU** | 91 | 91 | 65 | 11523 | 91 | 9641 | 91 |
|  | **ID** | Acaulospora | Acaulospora | Glomus | Entrophospora | Acaulospora | Entrophospora | Acaulospora |
|  | **%** | 8.5% | 11.3% | 12.0% | 8.1% | 5.8% | 10.8% | 6.6% |
| **3** | **OTU** | 11523 | 293 | 91 | 91 | 11523 | 91 | 2723 |
|  | **ID** | Entrophospora | Acaulospora | Acaulospora | Acaulospora | Entrophospora | Acaulospora | Glomus |
|  | **%** | 6.0% | 7.4% | 9.7% | 6.0% | 5.7% | 6.8% | 4.3% |
| **4** | **OTU** | 2723 | 259 | 11523 | 9641 | 2723 | 11523 | 11523 |
|  | **ID** | Glomus | Acaulospora | Entrophospora | Entrophospora | Glomus | Entrophospora | Entrophospora |
|  | **%** | 3.3% | 5.2% | 6.7% | 4.1% | 4.4% | 5.0% | 3.9% |
| **5** | **OTU** | 599 | 2723 | 1593 | 2723 | 10579 | 2723 | 9641 |
|  | **ID** | Paraglomus | Glomus | Gigaspora | Glomus | Entrophospora | Glomus | Entrophospora |
|  | **%** | 2.9% | 4.4% | 4.4% | 3.9% | 3.5% | 4.5% | 3.2% |

Supplementary Table 8. Taxonomic classifications of OTUs at the Genus/Species level and which were assigned to a functional group.

| Assigned Guild | Phylum | Class | Order | Family | Genera | Species |
| --- | --- | --- | --- | --- | --- | --- |
| AM fungi | Glomeromycota | Glomeromycetes | Diversisporales | Acaulosporaceae | *Acaulospora* | *morrowiae* |
|  | Glomeromycota | Glomeromycetes | Archaeosporales | Ambisporaceae | *Ambispora* | *leptoticha* |
|  | Glomeromycota | Glomeromycetes | Archaeosporales | Archaeosporaceae | *Archaeospora* | sp. |
|  | Glomeromycota | Glomeromycetes | Diversisporales | Gigasporaceae | *Cetraspora* | *pellucida* |
|  | Glomeromycota | Glomeromycetes | Glomerales | Claroideoglomeraceae | *Claroideoglomus* | *etunicatum* |
|  | Glomeromycota | Glomeromycetes | Diversisporales | Diversisporaceae | *Corymbiglomus* | *tortuosum* |
|  | Glomeromycota | Glomeromycetes | Diversisporales | Gigasporaceae | *Dentiscutata* | *heterogama* |
|  | Glomeromycota | Glomeromycetes | Diversisporales | Diversisporaceae | *Diversispora* | *aurantia* |
|  | Glomeromycota | Glomeromycetes | Diversisporales | Diversisporales | *Entrophospora* | sp. |
|  | Glomeromycota | Glomeromycetes | Glomerales | Glomeraceae | *Funneliformis* | *mosseae* |
|  | Glomeromycota | Glomeromycetes | Diversisporales | Gigasporaceae | *Gigaspora* | *margarita* |
|  | Glomeromycota | Glomeromycetes | Glomerales | Glomeraceae | *Glomus* | *versiforme* |
|  | Glomeromycota | Glomeromycetes | Diversisporales | Pacisporaceae | *Pacispora* | *scintilla* |
|  | Glomeromycota | Glomeromycetes | Paraglomerales | Paraglomeraceae | *Paraglomus* | sp. |
|  | Glomeromycota | Glomeromycetes | Diversisporales | Gigasporaceae | *Racocetra* | *fulgida* |
|  | Glomeromycota | Glomeromycetes | Diversisporales | Diversisporaceae | *Redeckera* | *fulvum* |
|  | Glomeromycota | Glomeromycetes | Glomerales | Glomeraceae | *Rhizophagus* | sp. |
|  | Glomeromycota | Glomeromycetes | Diversisporales | Gigasporaceae | *Scutellospora* | sp. |
|  | Glomeromycota | Glomeromycetes | Glomerales | Glomeraceae | *Septoglomus* | *viscosum* |
|  |  |  |  |  |  |  |
| Insect Pathogens | Ascomycota | Sordariomycetes | Hypocreales | Ophiocordycipitaceae | *Ophiocordyceps* | *sinensis* |
|  | Ascomycota | Sordariomycetes | Hypocreales | Clavicipitaceae | *Paecilomyces* | *carneus* |
|  | Ascomycota | Sordariomycetes | Hypocreales | Clavicipitaceae | *Paecilomyces* | *marquandii* |
|  | Ascomycota | Sordariomycetes | Hypocreales | Clavicipitaceae | *Paecilomyces* | *penicillatus* |
|  | Ascomycota | Sordariomycetes | Hypocreales | Clavicipitaceae | *Paecilomyces* | sp. |
|  | Ascomycota | Sordariomycetes | Hypocreales | Cordycipitaceae | *Beauveria* | *bassiana* |
|  | Ascomycota | Sordariomycetes | Hypocreales | Cordycipitaceae | *Beauveria* | *felina* |
|  | Ascomycota | Sordariomycetes | Hypocreales | Clavicipitaceae | *Metarhizium* | *anisopliae* |
|  | Ascomycota | Sordariomycetes | Hypocreales | Clavicipitaceae | *Metarhizium* | *flavoviride* |
|  | Ascomycota | Sordariomycetes | Hypocreales | Clavicipitaceae | *Metarhizium* | sp. |
